# The relationship between emotional intelligence and parental stress management during the Covid‐19 pandemic

**DOI:** 10.1002/brb3.2692

**Published:** 2022-07-18

**Authors:** Fatemeh Mohammadi, Mehrangiz ShoaaKazemi

**Affiliations:** ^1^ Department of Women's and Family Studies Faculty of Social Sciences and Economics Alzahra University Tehran Iran

**Keywords:** Covid‐19, emotional intelligence, parents, stress management

## Abstract

This study planned and conducted to investigate the relationship between emotional intelligence and parental stress management during the Covid‐19 pandemic. Taking into account the role of emotional intelligence parameters in stress management, this study aimed to show how a family can stand on its own feet and overcome the crisis safely. We used a descriptive correlational method. The statistical population of the study included all parents living in Tehran who underwent the stress of the Coronavirus in 2021. The statistical sample included 420 randomly selected parents. Goleman Emotional Intelligence Questionnaire and Stress Management and Coping Skills Questionnaire were used for data collection. Data analysis was performed using Pearson's Correlation Coefficient Test and stepwise regression method. The results showed that the stress response factor plays an important role in the increase of the emotional intelligence score (scale), and a positive and significant relationship was observed between them with 99% confidence. We found a positive and significant relationship between empathy and parental stress management parameters during the Covid‐19 pandemic with 99% confidence. There is a significant negative relationship between self‐motivation and parental stress management during the Covid‐19 pandemic with 95% confidence. Relaying on the findings of our study, we concluded that we can help families to manage the parental stress during the Covid‐19 pandemic by strengthening the empathy parameter of emotional intelligence and reducing premature and unmanaged sensitiveness.

## Graphical abstract

This study planned and conducted to investigate the relationship emotional intelligence and parental stress management during Covid‐19 pandemic. Taking into account the role of emotional intelligence parameters in stress management, this study aimed to show how a family can stand on its own feet and overcome the crisis safely.

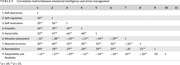

## 1 | INTRODUCTION

The outbreak of Coronavirus, which its origin remained unknown and subject of debate up to date of this paper, started from Wuhan, China, in late December 2019 and rapidly spread around the world. Since the production of an effective vaccine requires long production, testing, and confirmation processes, quarantine was introduced as the most effective way to break the Coronavirus chain (Wang et al., 2019). Afrashteh et al. (2020) introduced the traditional public health practices and quarantine as the only way to control the disease. However, when it comes to quarantine, stopping the normal routine of life, which in turn means a large number of people in the community have to stay home and endure the prolonged quarantine, will not pass without psychic and socio‐economic reactions (Khodabakhshi‐koolaee, 2020). The results of researches conducted in Canada on the psychological effects of quarantine revealed high prevalence of psychological distress and symptoms of stress and depression in the studied population (Hawryluck et al., 2004). When the stressor affects human life, our emotional state and physiological thinking system deviate from their normal and balanced order, the cognitive activities are affected, and some behavioral problems emerge in the form of feeling some degree of anxiety and depression (Reeve et al., 2013).

Family is one of the most important institutions and any disruption in its order not only affects the family members but also faces the community with serious problems (Minuchin, 2020). In a healthy and balanced family, there is an inherent ability and capacity to control stress and cope with crises. According to the psychologists, some of these stresses and crises in the family are expected and some other ones unexpected (Shafiabadi & Naseri, 2017). Researches have shown that if the couples can manage conflicts positively and have the ability to resolve them, such conflicts might not be harmful anymore (Zare, 2014).

A new concept has been introduced in recent years—what we call it emotional intelligence, which refers to the ability to identify and recognize the concepts and meanings of emotions, the relationships between them, reasoning, and problem solving. Highly emotionally intelligent people are more effective in coping with stressful events because they perceive and evaluate their emotions more accurately, they know when and how to express their emotions, and can regulate their moods in more effective way (Salovey & Mayer, 1990). The emotional intelligence helps people to feel that they can cope with difficult situations they encounter in their life (Goleman, 1995).

Therefore, in addition to paying attention to the personal and public health as preventive measures to get rid of the disease, it is necessary to pay special attention to the mental health of the community and, more especially, the family as its main building block. The mental healthcare, as an essential means in the world health system, will empower the people of the word in fighting, controlling, and eradicating the Coronavirus (Soklaridis et al., 2020). In a study conducted by Rezaei et al. (2020) entitled “The Effect of Home Quarantine due to Covid‐19 Disease Pandemic on Parental Stress and Its Relationship with Anxiety and Depression in Children of Gilani Province,” 181 subjects including children aged between 5 and 12 and their parents were studied. They found that the parents’ psychological impact has had devastating effects on children's depression, which makes the psychological support for parents and their children as an essential.

In a study by conducted by Spinelli et al. (2020) entitled “Parents’ Stress and Children's Psychological Problems in Families Facing the COVID‐19 Outbreak in Italy,” children aged between 12 and 14 took part in an online survey. They found that quarantine is a stressful factor for parents. This situation is accompanied with a more distressing experience for parents, which impairs their ability to provide care for their children. They suggested that “policies should take into consideration the implications of the lockdown for families’ mental health, and supportive interventions for the immediate and for the future should be promoted.”

According to the World Health Organization, mental health services have been stopped or disrupted in 93% of the countries around the world during the Corona pandemic where there is an increased demand for mental health services and support (Rogers et al., 2020). Considering that the issues raised about the importance of emotional intelligence as well as the role of the family and psychological factors during the coronavirus, here, a question arises: would we take more serious training of emotional intelligence in the light of its capabilities in managing emotions and coping with stressful events? What the present study seeks is to address this question: to what extent the parameters of emotional intelligence can manage the perceived parental stress during the Covid‐19 pandemic?

Studying stress without addressing the concept of coping and management makes a research incomplete and meaningless. Stress management is the actions that people take in response to stressors. These actions are either subjective or objective. Coping emphasizes the constant change in behavioral and cognitive efforts to manage the external and internal demands that are the source of the depletion of personal resources. Management includes efforts for minimizing, evading, tolerating, and accepting stressful situations (Jalali & Aghaei, 2015).

Clinical specialists of cognitive‐behavioral approach have made significant progress in formulating effective treatments for physical, mood, anxiety, sexual, and drug abuse. Their approach is applicable in groups, families, and individuals. In late 1979, cognitive‐behavioral therapy began its activity as part of group therapy and became the dominant treatment in the West. After that, Cognitive therapy of Beck Depression (1979) was described and developed (Nazari, 2015).

The most common stress management interventions are a combination of cognitive‐behavioral methods. They design a structure to reduce negative emotional arousals and correct incomplete cognitions of stressful situations. A review of the findings of stress management interventions shows that these interventions are effective in reducing psychological illness. It seems that this development in cognitive‐behavioral approach has an important role in designing therapeutic interventions (Mousavi, 2017).

## 2 RESEARCH METHOD

The method used in the present research is descriptive of correlation type. This study's statistical population includes all parents living in Tehran City who experienced stress during the Coronavirus pandemic in the year 1400 (starting from March 21, 2021). The studied statistical samples include 420 parents who were randomly selected using Cochran's formula. All parents had a diploma or a higher degree and had at least one child. The questionnaires were provided to one of the parents via online means. After collecting and eliminating incomplete questionnaires, the data were analyzed.

## 3 MEASURING TOOLS

1. Goleman Emotional Intelligence Questionnaire: The Emotional Intelligence Test is organized in the form of a five‐point Likert scale. The five‐point Shrink Emotional Intelligence Test measures five components of emotional intelligence (self‐awareness, self‐regulation, self‐motivation, empathy, and social skills). Each question indices a situation of the life that the subject must put him/herself in that position and select one of the options that is more compatible with his/her mental and psychological situation. In the present study, the reliability coefficient of this questionnaire was .81 by Cronbach's alpha method.
2. Coping Skills Inventory Questionnaire: This questionnaire consists of 42 items and the answers to its items are based on a five‐point Likert scale. The questionnaire consists of four factors, namely situation assessment, response to stress, remediation, and adaptability and flexibility which has high validity and reliability. In a study entitled “The Investigation of Relationship between Stress Coping Skills and Students’ Academic Achievement,” the researcher obtained the reliability of the whole test as .77 (Khayyer & Saif, 2004). In the present study, the reliability coefficient of this questionnaire was .79 by Cronbach's alpha method.

## 4 REVIEWING THE FINDINGS

In the descriptive analysis of the data, statistical indicators including mean and standard deviation and graphs and tables related to each of the research variables were examined.

As shown in Table 4, the mean total emotional intelligence is 101.03, the standard deviation is 15.29, its maximum is 148.00, and its minimum is 45.00. The mean self‐awareness is 26.50, the standard deviation is 4.33, its maximum is 40.00, and its minimum is 15.00. The mean self‐regulating is 20.94, the standard deviation is 4.60, its maximum is 34.00, and its minimum is 7.00. The mean self‐motivation is 21.24, the standard deviation is 4.68, its maximum is 33.00, and its minimum is 8.00. The mean empathy is 19.26, the standard deviation is 3.09, its maximum is 29.00, and its minimum is 10.00. The mean social skills is 13.08, the standard deviation is 3.77, its maximum is 25.00, and its minimum is 5.00. The mean stress management is 138.76, the standard deviation is 13.61, its maximum is 197.00, and its minimum is 47.00. The mean situation assessment is 62.44, the standard deviation is 9.60, its maximum is 85.00, and its minimum is 20.00. The mean reaction to stress is 25.90, the standard deviation is 5.87, its maximum is 43.00, and its minimum is 9.00. The mean remediation is 11.52, the standard deviation is 2.12, its maximum is 20.00, and its minimum is 4.00. The mean adaptability and flexibility is 17.16, the standard deviation is 2.83, its maximum is 24.00, and its minimum is 6.00.

## 5 FINDINGS

Based on the results of Table 1, among the parents took part in the study, 317 women and 103 men completed the questionnaires. The average age of women was 38.79 with a standard deviation of 7.91, and the average age of men was 39.43 with a standard deviation of 8.09.

**TABLE 1:** Gender frequency distribution related to the participants of the questionnaires

Variable	Groups	Frequency	Percent
Group	Female	317	75.5
	Male	103	24.5
	Total	420	100.0

Based on the results of Table 2, the education levels of the women participating in the study were as follows: 98 with high school diploma, 146 undergraduate degrees, 62 master's degree, and 11 ones with holding PhD degrees. The education levels of men participating in the study were as follows: 9 with high school diploma, 32 with undergraduate education, 49 with master's degrees, and 13 holding PhD degrees.

**TABLE 2:** Frequency distribution of education by gender

Total	PhD	Master	Bachelor	Diploma	Groups	Variable
317	11	62	146	98	Female	Group
103	13	49	32	9	Male	
420	24	111	178	111	Total	

Based on the results of Table 3, the number of children of the participants in the study: 189 families with one child, 131 families with two children, 60 families with three children, 11 families with four children, 1 family with five children, and the number of children in 28 families was not specified.

**TABLE 3:** Frequency distribution table of the number of children of the parents

Percent	Frequency	Groups	Variable
45	189	One child	Number of children
31.2	131	Two children	
14.3	60	Three children	
2.6	11	FOUR children	
0.2	1	five children	
6.7	28	Not specified	
100.0	420	Total	

**TABLE 4:** Mean and standard deviation of research variables

Variables	Components	Minimum	Maximum	Mean	Standard deviation (SD)
Emotional intelligence	Self‐awareness	15.00	40.00	26.501	4.331
	Self‐regulating	7.00	34.00	20.945	4.606
	Self‐motivation	8.00	33.00	21.247	4.687
	Empathy	10.00	29.00	19.264	3.091
	Social skills	5.00	25.00	13.081	3.770
	Total score	45.00	148.00	101.039	15.292
Stress management	Situation assessment	20.00	85.00	62.44	9.60
	Reaction to stress	9.00	43.00	25.90	5.87
	Remediation	4.00	20.00	11.52	2.12
	Adaptability and flexibility	6.00	24.00	17.16	2.83
	Total score	47.00	197.00	138.76	13.61

As shown in Table 5, there is a significant negative relationship between situation assessment and self‐regulation, self‐motivation, empathy, social skills, and overall emotional intelligence at *p* < .01 level. There has been no significant relationship between situation assessment and self‐awareness. There is a positive and significant relationship between reaction to stress and self‐awareness, self‐regulation, self‐motivation, empathy, social skills, and the overall score of emotional intelligence at *p* < .01 level. There is no significant relationship between remediation, self‐awareness, and overall emotional intelligence. There is negative and significant relationship between adaptation and flexibility, self‐awareness, self‐regulation, self‐motivation, empathy, social skills and overall emotional intelligence at *p* < .1 level. There is a negative and significant relationship between stress management and self‐motivation at *p* < .05 level. There is a positive and significant relationship between stress management and empathy at *p* < .01 level. No significant relationship was found between self‐awareness, self‐regulation, and social skills with overall emotional intelligence score.

**TABLE 5:** Correlation matrix between emotional intelligence and stress management

	**1**	**2**	**3**	**4**	**5**	**6**	**7**	**8**	**9**	**10**	**11**
1. Self‐awareness	1										
2. Self‐regulation	.34**	1									
3. Self‐motivation	.35**	.56**	1								
4. Empathy	.36**	.39**	.45**	1							
5. Social skills	.33**	.57**	.66**	.48**	1						
6. Situation assessment	−.07	−.38**	−.43**	−.11**	−.44**	−.39**	1				
7. Reaction to stress	.20**	.40**	.52**	.30**	.53**	.52**	−.26**	1			
8. Remediation	.049	.18**	.19**	.18**	.29**	.23**	−.20	.51**	1		
9. Adoptability and flexibility	−.12**	−.27**	−.39**	−.12**	−.34**	−.34**	.65**	−.20**	.05	1	

* *p* < .05, ** *p* < .01.

According to Table 6, in the first step, the empathy variable was analyzed and explained 1% of the variance in stress management. In the second step, after addition of self‐motivation, the value of determined variance increased from 1% to 4%.

**TABLE 6:** Summary of regression model

Model	*R*	*R* ^2^	*R* ^2^ (adjusted)	Standard estimation error	Durbin‐Watson Table
Step one	.128	.01	.01	13.52	
Step Two	.218	.04	.04	13.32	1.98

According to the results of Table 7, And F results, and also according to the significance level of regression, stress management prediction based on emotional intelligence subscales at the level of .01 is significant.

**TABLE 7:** Significance of regression model for stress management based on emotional intelligence subscales

	Source of variation	Sum of squares (SS)	Degree of freedom (DF)	Mean square (MS)	F	*p*
Step One	Regression	1263.37	1	1263.37	6.90	.001*
	Residual	76,277.74	417	182.92		
	Total	77,541.12	418			
Step two	Regression	3683.12	2	1841.56	10.37	.001*
	Residual	73,858.00	416	177.54		
	Total	77,541.12	418			

* p < 0.01.

Based on the results of Table 8, in step one, empathy is significant for prediction of stress management at *p* < .1 level. In step two, by addition of self‐motivation to empathy, the self‐motivation is also significant in prediction of stress management at *p* < .01 level.

**TABLE 8:** Regression coefficients of emotional intelligence subscales in the whole sample

	Coefficients	Nonstandardized coefficients effect, B	Standardized coefficients effect, β	T	*p*
Step one	Fixed	127.91	–	30.673	.001
	Empathy	.562	.128	2.628	.009*
Step two	Fixed	132.611	4.301	–	.001
	Empathy	.950	.216	4.036	.001*
	Self‐motivation	−.573	−.197	−3.692	.001*

* p < 0.01.

The standardized regression effect coefficient or beta (β) coefficients of empathy was .128 and self‐motivation was −.197. This means that the 1 increase in standard deviation of empathy, .128, will be added to the stress management score, and 1 increase in standard deviation of self‐motivation, −.197, will be decreased from stress management score.

The regression equation can be calculated using the nonstandardized coefficients column as follows:

$$\begin{array}{ccc} \left(Self-motivation\right)\left(-0.197\right) & + & \left(empathy\right)\left(0.128\right)+127.91\\ & & =stress\;management \end{array}$$

## 6 DISCUSSION AND CONCLUSION

The present study aimed to investigate the relationship between emotional intelligence and parental stress management during the Covid‐19 pandemic. Based on findings of our study, from among of the four stress coping skills, there was a positive and significant relationship between only one skill—stress response and all components of emotional intelligence as well as its overall score. This may imply that reaction to stress may be the first step in managing parental stress during the Coronavirus pandemic. From the point of view of two stress coping strategies, the reaction to stress is emotion oriented, and in the other three ones are problem oriented. According to the findings of our study, during the Coronavirus pandemic, the emotion‐oriented coping strategy, which aims at quick reduction or relief the situation, was more effective than the problem‐oriented approach, which simply aims on overcoming the situation and changing the source of stress.

Also, a positive and significant relationship was observed between remediation skill and the self‐regulation, self‐motivation, empathy, and social skills variables, but there is no significant relationship between this skill and self‐awareness and the overall score of emotional intelligence. In regard of ineffectiveness of the self‐awareness variable, it may be necessary to change the image that the parental perceives form it as a family. The additional load that a mother experiences because of children staying at home and managing their education needs during Coronavirus may shift her focus from her husband to children. The focus on the education of the children is quite different from the result of education efforts. When the women's priority shifts from a wife's role to a mother's role, the boundaries will be also shifted accordingly.

Also, the relationship between situation assessment skills and parameters like self‐regulation, self‐motivation, empathy, social skills, and the overall score of emotional intelligence was negative and significant. The existence of a negative and significant relationship in this study may be a function of the fact that although emotion‐oriented confrontation avoids facing the reality of the problem and is a temporary solution, every problem could not be resolved easily and the one's mental health may be affected without a temporary and short‐term calm. Failure to successfully use stress coping techniques can also lead to fatigue and emotional exhaustion and may have negative effect on the emotional intelligence score. On the other hand, the family will be isolated from the rest of the community despite its communicative nature. As a result, the family, as the most important subset of the social system, will distance itself from the community. The commonalities and a common social concern that the family members, especially the parents perceive, prevent them from getting involved in individual and minor issues and lead them towards solving social problems. This process will lead the family to pursue its goals at a higher level and organize its performance in such a manner to achieve such higher goals. If the family does not have a problem, then it will itself become a problem. When the family takes a step beyond individualism in favor of the community, it will surely play a positive role in resolving social problems.

However, no significant relationship was observed between situation assessment skills and self‐awareness parameters. During the Corona pandemic that the parental received different, and sometimes exaggerated, messages from the authorities, this may impair the self‐awareness of the subjects that took part in our study and made this parameter as an ineffective factor. The problem‐oriented skills such as situation assessment, adaptability, and flexibility may need activation of higher level mental processes than other coping skills at the time of facing with stressful situations like Coronavirus pandemic. In other words, it may be necessary to first reach a more stabilized mental state in order to have the opportunity to activate one's mental processes.

Also, the present study confirmed the hypothesis of existence of a relationship between the self‐motivation of emotional intelligence and parental stress management, and a significant negative relationship was observed. The results of research on the Coronavirus crisis also showed that the rate of domestic violence has increased, which indicates an increase in sensitiveness among family members which in turn can have adverse effect on parental stress management. The undesirable levels of sensitiveness during the Coronavirus may be a function of spending long hours at home and the resulting anxiety along with economic and psychological problems which results in emergence of tension between the parents.

Helping others enhances family ties and strengthening the empathy will enhance our ability to manage emotions in stressful situations. In the present study, a significant positive relationship was also observed between the empathy and stress management parameters. There is no contradiction between the findings of studies conducted by Hafizi et al. (2011), Barani (2017), Soleimani and Alibeygi (2009), and the results of our study. On the other hand, Goleman believes that the observation of self‐control is not an easy; the lower the severity of emotions, the better the self‐control. Findings of Hosseinmardi et al. (2016), Iranban (2017), Heydari Tafreshi and Delfan Azari (2010), and Abdoli et al. (2012) are consistent with the results of this study.

As the results of the present study indicates, the imbalanced combination of emotional intelligence parameters description above has had effect on the overall score of emotional intelligence and, contrary to the research hypothesis, emotional intelligence was not able to manage the parental stress during the Covid‐19 pandemic. Although the emotional intelligence can be helpful in parental stress management, instability in the family may impair the ability of people with moderate emotional intelligence to use their emotional intelligence skills and may not be able to use their usual emotional knowledge as well. Given the timing of the research and the instability of the family structure during the Coronavirus, perhaps, a significant portion of the lack of emotional intelligence has been the result of the above‐mentioned problems. The more stable the family structure, the more productive the emotions. Of course, it is also a possible event that a family, which is in direct path of development, may experience some problems when encounters with a specific crisis. The unexpected performance of the family in such situation tell us that the hidden rules regulating the family ties may become ineffective or inappropriate and needs to be discussed again. Sometimes, this approach may be ineffective merely due to resistance and/or noncooperation of one of members of the family. Also, one of the most important explanations and examples of insignificance of emotional variables in stress management is the issue of severe conflict and separations in marital life during the Corona pandemic which the society is still witnessing and occurs in different social classes. Although the findings of this study are consistent with the results of researches conducted by Fooladvand et al. (2015), Abdoli et al. (2012), Vancouver et al. (2002), inconsistency between finding of this study and some other ones may be due to the time of the study. As far as we reviewed, all researches have been conducted in a situation where the family has not faced with social stress, but the present study was conducted in a situation that the families were experiencing abnormal stress; therefore, we cannot compare the results of this study with the results of previous researches.

The findings of this study revealed that event the emotional intelligence is a necessary condition for the optimal functioning of the parents, but it is not the sufficient condition. Therefore, considering the different nature of Coronavirus pandemic, we suggest the role of other psychological interventions in parental stress management as well as social factors like performance of media and authorities, and National Headquarter for Coronavirus should be investigated as well.

The present study, like other studies, has some limitations. Lack of resources, the method of conducting the research, and the procedure of data collection, which was done online, are considered as limitations of this study. The absence of the researchers at the time of completing the questionnaires by the participants in order to answer their questions was another limitation of the present study. Considering the role of cultural and geographical factors, it should be noted that this research has been conducted in Tehran and generalizing it to other cities should be done with cautious. Awareness, attitudes, stress, anxiety, and the need to perceive psychological care of people from other countries may differ from the findings of the present study. Also, the limitations related to the statistical population, type of the research, generalization of findings, interpretation of results, as well as problems related to the Coronavirus period should not be overlooked in this study.

7

### PEER REVIEW

The peer review history for this article is available at https://publons.com/publon/10.1002/brb3.2692

## Data Availability

Data are accessible from authors on reasonable request.
